# An Update of the Possible Applications of Magnetic Resonance Imaging (MRI) in Dentistry: A Literature Review

**DOI:** 10.3390/jimaging7050075

**Published:** 2021-04-21

**Authors:** Rodolfo Reda, Alessio Zanza, Alessandro Mazzoni, Andrea Cicconetti, Luca Testarelli, Dario Di Nardo

**Affiliations:** Department of Oral and Maxillofacial Sciences, Sapienza University of Rome, 00161 Rome, Italy; rodolforeda17@gmail.com (R.R.); ale.zanza@gmail.com (A.Z.); alessandro.mazzoni@uniroma1.it (A.M.); andrea.cicconetti@uniroma1.it (A.C.); dario.dinardo@uniroma1.it (D.D.N.)

**Keywords:** magnetic resonance, imaging, MRI, dentistry, endodontics, implantology, maxillary sinus, dental materials, CBCT

## Abstract

This narrative review aims to evaluate the current evidence for the application of magnetic resonance imaging (MRI), a radiation-free diagnostic exam, in some fields of dentistry. Background: Radiographic imaging plays a significant role in current first and second level dental diagnostics and treatment planning. However, the main disadvantage is the high exposure to ionizing radiation for patients. Methods: A search for articles on dental MRI was performed using the PubMed electronic database, and 37 studies were included. Only some articles about endodontics, conservative dentistry, implantology, and oral and craniofacial surgery that best represented the aim of this study were selected. Results: All the included articles showed that MRI can obtain well-defined images, which can be applied in operative dentistry. Conclusions: This review highlights the potential of MRI for diagnosis in dental clinical practice, without the risk of biological damage from continuous ionizing radiation exposure.

## 1. Introduction

The purpose of this narrative review was to evaluate the current evidence for the application of magnetic resonance imaging (MRI), a radiation-free diagnostic exam, in some fields of dentistry.

Radiographic imaging plays a significant role in the current first and second level of dental diagnostics and treatment planning [1,2]. With the introduction of cone beam computed tomography (CBCT), three-dimensional imaging prescription has become common in orthodontics, periodontology, implantology, and endodontics, with dedicated software becoming increasingly useful in these specific disciplines [3]. The merits of CBCT in treatment planning over conventional two-dimensional radiographic imaging are remarkable. However, the main disadvantage is the high exposure to ionizing radiation for patients, which does not allow clinicians to repetitively use this type of examination in a short window of time, with a consequent need for a careful assessment of the expected risk/benefit ratio in each individual case [4]. 

Three-dimensional images of the maxillofacial area are currently acquired by computed tomography (CT), cone beam computed tomography (CBCT), and magnetic resonance imaging (MRI) devices. 

MRI, a well-established imaging technique in various areas of medicine, has become fundamental for the non-invasive diagnosis of soft tissue diseases since it has the great advantage of not using ionizing radiation, avoiding the biological damage related to the other three-dimensional imaging techniques such as CT and CBCT. MRI is almost comparable to the latter in terms of spatial resolution and data visualizing ability in the visions of the transverse and panoramic planes, which are most familiar to dentists [5].

For years, in dentistry, MRI imaging has always found a great application in the diagnosis of temporomandibular disorders (DTM) due to tissue histology, which has characteristics that perfectly match the type of diagnostic examination [6].

Therefore, considering the great amount of literature published regarding TMJ imaging, and since this diagnostic exam has been included in the guidelines for a long time, this review was focused on its applicability to the other branches of dentistry, which, to date, have not given great importance for this type of imaging technique [7]. Furthermore, since MRI does not use ionizing radiation, it is particularly relevant for repeated examinations in children [4].

Unlike radiographic imaging, the MRI technique is based on the presence of a magnetic field, formed by an MRI scanner in which the patient is positioned. The images are generated by measuring “signals” sent back by protons excited by the magnetic field, in particular by hydrogen atoms. Precisely for this reason, a better visualization of tissues containing water, such as the human brain, is obtained [8].

MRI creates the images using a strong and uniform static magnetic field and radio frequency pulses. When placed in a magnetic field, all substances are magnetized to a degree that depends on their magnetic susceptibility. Unfortunately, variations in magnetic field strength occurring at the interface between dental materials and adjacent tissues can lead to spatial distortions and signal loss, thus generating artifacts in the images [9].

In addition to the formation of artifacts, other undesirable effects of MRI could be radiofrequency, physical effects such as heating, and magnetically induced displacement (a mechanical effect) of dental materials [9].

Its application to dentistry, considering its risk–benefit ratio, would make it a very interesting exam. In particular, this review was focused on the comparison of the role of MRI in different branches of dentistry: endodontics, oral and maxillofacial surgery, and implantology. If RMI examination, to date, has not been considered enough as a diagnostic aid, the reason could be found in the disadvantages deriving from its application.

However, the high acquisition costs and long scan times are the main, and more discussed, disadvantages of the MRI technique. In addition, the risk that the patient suffers from claustrophobia, or that the patients have biomedical devices such as pacemakers, cochlear implants, neurostimulators, or infusion pumps represents an important contraindication; fixed metal prostheses and aneurism clips, to date, no longer represent a contraindication.

**Figure 1:**
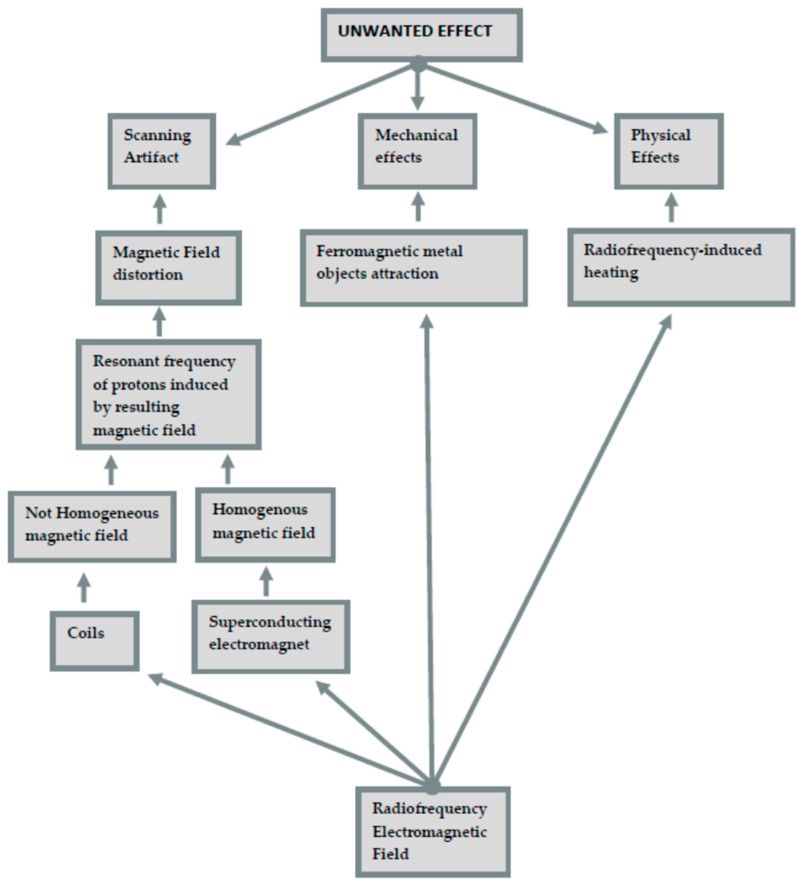
Influence of an electromagnetic field.

Chockattu et al. have clearly exposed the undesirable effects of magnetic resonance imaging [9]. The undesirable effects that are caused by the interaction of MRI and dental materials fall into three broad categories:

1. Scanning artifact. These artifacts are defined by pixels that do not faithfully represent the tissue components studied. The shape of these artifacts depends on the scanning plane, whether it is axial or sagittal. The severity depends on the magnetic properties and position of the present metal; its orientation, shape, number; the homogeneity of the alloy; and the MRI sequence used. On this topic, the literature contains contradictory results, depending on where the attention has been focused on, whether that was gold content alloys, titanium, or a dental amalgam [9,10,11,12]. Distortion of the static magnetic field is generated from the difference in the magnetic susceptibility, as signal incoherence is generated by substances with different magnetic capacities.
  In addition to this typology, there are also artifacts caused by eddy currents, induced by alternating gradients and radiofrequency magnetic fields, which participate in generating distortions.
2. Mechanical effects (magnetically induced displacement). The most immediate risk associated with the MR environment is the attraction between the MRI device (a magnet) and ferromagnetic metal objects. The magnetic field is strong enough to pull heavy objects towards the scanner at a very high velocity, this is also known as “the projectile effect”. Patients at the highest risk are those with metals not belonging to medical devices (e.g., projectiles, piercings, welding droplets), and among patients with medical devices, those with pacemakers, cochlear implants, neurostimulators, and infusion pumps are at risk.
  The complications related to RMI can cause malfunction, dislocation, and soft tissue burns (due to the absorption of radiofrequency energy).
3. Physical effects (radiofrequency heating). Metallic objects in the human body, such as pacemakers, cochlear implants, neurostimulators, and infusion pumps, before human tissues themselves, can undergo radiofrequency-induced heating. In addition, the batteries of medical devices can also be subject to rapid discharge.
  Unwanted effects and the mechanisms that generate them are shown in Figure 1.

Chockattu et al. emphasize the relevance of factors influencing unwanted effects, such as magnetic susceptibility and magnetic permeability. The susceptibility represents a measure of the extent to which a substance becomes magnetized when it is placed in an external magnetic field. The greater the magnetic permeability (and so, alloy composition) of a material, the more magnetic field distortion it will produce [13]. 

During MRI, the magnetic field could also be distorted by electric currents, due to electrical conductivity, flowing in materials within or close to the machine. These currents are induced in materials by a fluctuating magnetic field (summarized in Figure 1).

The magnetic permeability and tensile strength are linked by a relationship of direct proportionality. The tensile strength depends on the crystalline structure of the metal, so the past mechanical history of stainless-steel alloys determines their future effect on MRI images.

Moreover, the MRI sequence can also influence artifact formation; in effect, some sequences are more sensitive than others [14].

Dental materials, used in different dental procedures, which can often generate artifacts, do not include the different dental tissues, which do not generate artifacts.

There are many materials used in dentistry that can affect the quality of the MRI examination, which, in the past, represented a reason for not prescribing this diagnostic exam [10,12]. Endodontic materials such as resin-based sealer and gutta-percha seem not to produce detectable distortions on MRI [5,9]. In fixed orthodontic treatment, NiTi arch wires and stainless-steel brackets can distort local magnetic fields, causing large artifacts and making image interpretation very difficult [13,15,16]. Regarding maxillofacial prostheses, ferromagnetic devices should ideally be removed. Dental implants are made of titanium, a non-ferromagnetic material, and of ferromagnetic iron, which causes a drop-out of signal, causing artifacts [17]. 

In restorative dentistry, some materials seem to produce undetectable distortion on MR imaging, such as glass-ionomer cements and composite resins [5]. Polycarboxylate, zinc phosphate-based cement, and some modified dimethacrylates can also produce small image artifacts [18].

The amalgam, not frequently used today, that represented the gold standard in conservative dentistry until 20 years ago, is composed from several metals (copper, silver, tin, zinc, palladium, platinum, and mercury), with silver as the major component. Silver is a non-ferromagnetic metal, and so it does not have a significant influence in dental MRI. 

In prosthetic dentistry, gold crowns are relatively free of ferromagnetic effects, due exclusively to traces of other metals that pollute the alloy. The ability to generate distorting ferromagnetic effects is very limited. Ceramic and zirconia crowns do not generate any artifacts, but, regarding zirconia, there are conflicting studies, with some comparing its effects to those of metals [19,20]. Metal–ceramic restorations, frequently with nickel alloys, seem to show a tendency to generate artifacts [9,15]. Dental materials that generate artifacts are summarized in Table 1. It is also important to underline that not only the material, but also the shape of the metal object affects the quantity of artifacts: an arch- or ring-shaped implant will extinguish the signal inside the ring/arch, generating any artifacts.

## 2. Materials and Methods

A search for articles on dental MRI was performed using the PubMed electronic databases. The following keywords (Magnetic Resonance Imaging, MRI, Implantology, Endodontics, Periapical Lesions, Anatomy, Artifacts, Maxillary Sinus) combined with several Boolean operators were searched.

Five hundred and twenty-five articles were screened, and only 37 studies were included. According to the authors, only some articles about endodontics, conservative dentistry, implantology, and oral and craniofacial surgery that best represented the aim of this study were selected. The articles selected and not related to these branches of dentistry were considered only for the technical specifications and considerations on the functioning of MRI. Original research articles on gnathology and joint disorders, orthodontics, and prosthetics not concerning the topics listed above were excluded.

## 3. Results

**Figure 2:**
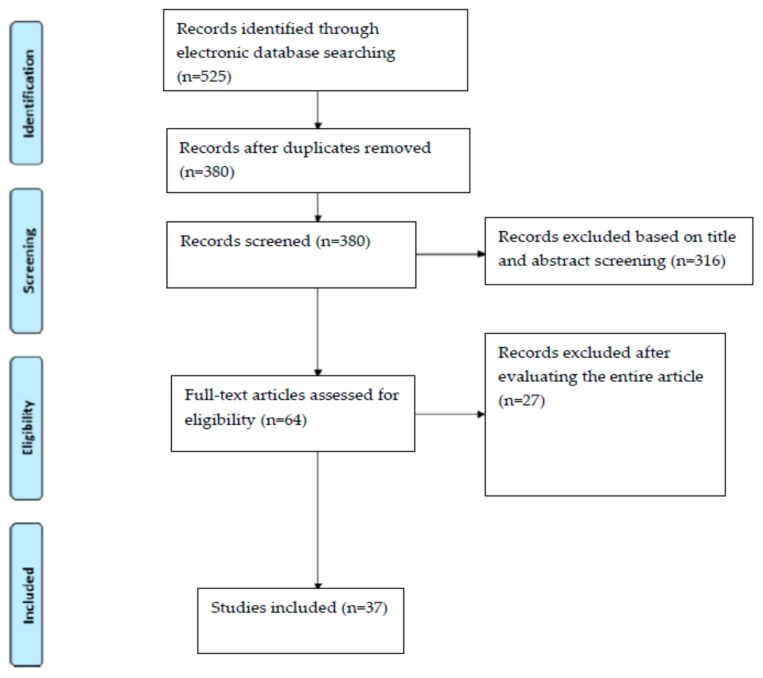
Selection process for the studies included (following PRISMA Statement) [21].

All the included articles showed that MRI can obtain well-defined images, which can be applied in operative dentistry. The studies’ selection flow-chart is represented in Figure 2; studies that passed the inclusion criteria and were considered for review are shown in Table 2. In the right conditions, with proper attention to teeth, bone, and the tissues of the maxillofacial region, MRI can offer very important information not easily obtainable with other diagnostic exams. Dental MRI can also recognize pathological endodontic conditions such as decay, microcracks, and necrotic pulp tissues. Moreover, it can diagnose periapical granulomas from a cystic lesion and can represent an important aid in maxillary sinus conditions diagnosis and in implant surgery planning.

## 4. Discussion

### 4.1. Fundamental Parameters in MRI

In dental MRI, signal-to-noise ratio (SNR) and resolution are two fundamental parameters to be considered; SNR is measured by calculating the ratio between the signal intensity in an area of interest and the standard deviation of the signal from the background [5]. The image resolution depends on the image voxel size. In MRI, the SNR can be improved by decreasing the matrix size, increasing the voxel size, increasing the field of view (FOV), reducing the bandwidth using surface coils, increasing the slice thickness, using an echo time (TE) of spin echo sequence as short as possible, and increasing the number of signal acquisitions (NA) [5,19].

The more the SNR is increased with the above-mentioned actions, the more the images’ definition decreases.

To reduce the FOV with the aim of increasing the resolution of the images without reducing SNR, it is necessary to use dedicated coils. The commonly used head or neck coils cannot reach an optimal resolution for being applied to improve dental diagnoses; intraoral positioning of the coil may increase both the resolution and the SNR, but it is very difficult to use due to anatomical limitations [9,19]. One of the most comfortable coil positions was proposed by Idiyatullin et al., with the advantages of using a loop coil, very similar to an impression tray, in the occlusal position for dental applications [22].

Despite the progress obtained so far, with the purpose of optimizing these parameters, the results (discussed below) are positive.

### 4.2. Apical Periodontitis Diagnosis

Regarding apical periodontitis, it is a chronic inflammatory disease of peri-radicular tissues, usually caused by a chronic bacterial infection of the root canal system near the bone. The pathogenesis of apical periodontitis and the cause of endodontic failure have been extensively reviewed by Siqueira and by Nair: the main role is played by bacteria (mainly obligate anaerobes and fungi), depending on the relationship with the host’s immune system [23,24]. Endo-osseous development of these conditions prevents the arrival of immunity cells and antibiotic molecules through the bloodstream. In order to be radiographically visible with bidimensional RX, a periapical radio-lucency should reach from 30% to 50% of bone mineral loss [25].

Sometimes these lesions heal spontaneously, sometimes they get worse, so much so that they enlarge and compress noble structures, or pour out as abscesses outside the bone [23,26]. 

The chronicity of these lesions makes them capable of corroding the bone in their proximity, visible radiographically as radiolucent lesions, although histological studies show that they can differ between granuloma or cyst [26,27,28].

Nair showed that up to 85% of all periapical lesions are granulomas [24].

Periapical granulomas contain granulomatous tissue, cell infiltrates, and a fibrous capsule, root cysts are considerably less frequent, and occur in two distinct histological categories: true apical cysts and pocket apical cysts [28,29]. True root cysts are entirely enclosed by the cyst wall epithelium, developed from the dormant epithelium, also known as epithelial rests of Malassez, after local inflammation stimuli. Periapical pocket cysts are lined by the epithelium but are open to the root canal, effectively isolating a pocket-like micro abscess from the periapical environment. This division is not merely histological, but has an important influence on the treatment, as the chances of recovery are very different from one to the other: granulomas and pocket cysts can heal after orthograde root canal therapy, while true cysts are self-sufficient and therefore less likely to be resolved with non-surgical treatment, hence, without removal of the cystic epithelium [26,28]. In the literature there are different opinions on this topic, not all authors agree about this definition [29]. Furthermore, larger lesions (more than 5 mm) are more likely to be root cysts associated with lower success rates for orthograde treatment [26,30].

From these considerations, the need for a diagnostic exam is highlighted, such as MRI, free from biological damage, unlike CBCT or CT, and able to evaluate in vivo the nature of the lesion and to orient the clinician towards the most appropriate treatment, whether it is surgical for true root cysts or endodontic, orthograde retreatment for periapical pocket cysts or granulomas [31].

One of the main advantages of MRI over CT and CBCT is the high soft tissue contrast and the ability to vary the contrast by changing the design of the MRI sequence, as well as the absence of ionizing radiation [30].

More specifically, MRI not only provides excellent soft tissue contrast but also allows for the evaluation of specific tissue components in different sequences.

Given these strengths, MRI has shown diagnostic superiority over CT techniques in various soft tissue associated pathologies in the head and neck region, in fact, MRI is the most suitable examination for the study of brain and solid tumors [9].

Therefore, surgical biopsy and subsequent histopathological evaluation remains the gold standard to confirm the diagnosis of different periapical lesions, but it obviously represents the most invasive technique considering the risk/benefit ratio. In this specific case, it is emphasized how this examination can simplify the diagnosis, having marked characteristics in evaluating lesions filled with liquid. Technical advances associated with the use of higher field strength, dedicated coil systems, and optimized sequencing techniques resulted in improved image quality, followed by increased interest in magnetic resonance imaging in dentistry [27,32].

To date, however, only Geibel and colleagues have systematically analyzed apical bone lesions with MRI; in a comparison between MRI and CBCT for the diagnosis of periapical lesions, they concluded that MRI is useful for the identification of fluids (hypointense T1-weighted images and hyperintense on T2-weighted images) and fibrous tissue (isointense on T1- and T2-weighted images) [32].

MRI has shown greater sensitivity in diagnosing periapical lesions than CBCT, in particular, when cystic fluid was present, thus excluding that it may be a vascularized lesion, such as a peri-apical granuloma. Moreover, it can more precisely diagnose the true dimensions of a lesion, and can provide a better estimation of the relationship between a lesion and critical structures, such as nerves and vessels [30].

Granulomas, on the other hand, are very heterogeneous due to the chronical infiltration of different immunity cells. Another important differentiation is represented by the wall of the lesion, with “thin-walled” cysts (mean: 1.6 mm) and “thick-walled” granulomas (mean: 4.6 mm), the latter also having poorly defined lesion margins in both MRI and in CBCT.

Moreover, the internal texture is very different; it is homogeneous in cysts, and inhomogeneous in granulomas [27,28,29,30,31,32]. Several authors postulated that dental MRI could detect inflammatory pathologies at an early stage, long before CBCT or conventional radiographs [14,30,32].

In many cases, the teeth on which these pathologies develop have already undergone primary endodontic treatment, and therefore the roots are reamed and filled with dense filling materials.

Geibel et al. believe it is very difficult to identify the root apex of the responsible tooth in these cases due to the presence of artifacts [32]. As previously stated, in the study of Chockattu et al., the same number of artifacts were not present with MRI, and when present, they appeared to produce undetectable distortions, unlike CBCT [9].

Therefore, it can be concluded that the MRI technique is essential for the analysis of periapical lesions, as these lesions must be adequately imaged with regard to resolution, contrast, signal-to-noise ratio, and susceptibility to artifacts.

### 4.3. Evaluation of Dental Fractures

Endodontically treated and incorrectly restored teeth, in addition to suffering more frequently from periapical infections, have a greater risk of fracturing [33]. 

Regarding dental fractures, MRI has the potential to help in determining the presence and extent of cracks and fractures in teeth due to good contrast, and especially without exposure to ionizing radiation as with CBCT, which is considered the current clinical standard [34].

In most cases, discontinuities cannot be definitively visualized in the absence of invasive measures such as CBCT imaging; in the study of Schuurmans et al., the aim was to develop MRI criteria for the identification of root cracks and fractures and to establish reliability and accuracy in their subsequent detection [5,34].

It is important to underline that these authors used in vivo MRI acquisition sequences on extracted teeth. A problem when evaluating these MRI studies is that in vitro sequences are frequently applied, with very long acquisition times, and they are impossible to be applied in vivo, and therefore are far from improving clinical practice. From the results of these studies, it is possible to highlight that MRI, thanks to the higher contrast, has allowed for better evaluation of cracks and fractures compared to CBCT imaging [34]. 

Part of these results is related to the reduced number of artifacts generated from radiopaque materials compared to CBCT imaging; this statement is very important because endodontically treated teeth that were root-filled are more prone to fracture if not correctly restored, due to tooth substance loss [9,10,33].

In conclusion, the advantages of contrast enhancement, and the absence or reduction of radiopaque material artifacts in MRI and comparable sensitivity and specificity measures with CBCT, suggest the importance of improvements in magnetic resonance quality, particularly in image acquisition and post-processing parameters. Always remembering the absence of ionizing radiation, and the continuous improvements that this imaging exam is obtaining, the next applications of dental MRI in detecting dental cracks or fractures may involve defining the minimum physical size for detection using advanced MRI sequences [12,18,19].

### 4.4. Endodontics, Endodontic Anatomy and Conservative Dentistry

Regarding endodontic anatomy, while performing an endodontic treatment, it is extremely important to create a correct and accurate topographic image of the root canal system; knowing the anatomy well before starting endodontic treatment allows the clinician to use the most suitable instruments in the correct way, avoiding subjecting them to considerable stress that could lead to intracanal separation [35,36,37,38].

Up to date, visualization of root canal topography and dental anatomy has been obtained by conventional bi-dimensional radiographs, and only in recent years has CBCT been increasingly applied, due to the reduction of the exposure dose, the increasing availability of the machinery in the private practice, and the reduced costs compared to the past or to other exams. MRI offers high-level tissue visibility, equal to or even greater than CT and CBCT, but it requires sufficient resolution that tends to be achieved only with much longer scan times, without, however, exposure to ionizing radiation.

Several articles have shown the usefulness of spin echo and gradient echo imaging, single point imaging, and SPRITE and STRAFI techniques for the visualization of tooth surface geometry, as well as for distinguishing between soft tissue and mineralized tissue in extracted teeth [38]. The high-intensity signal from water and the lack of signal from mineralized tissues produce a high contrast that allows for the recognition of the dental crown and the outline of the pulp chamber, root canals, and carious lesion [39,40,41].

Bracher et al. stated that carious tissues provide an intense signal, easily recognizable in the 3D reconstruction performed by the software.

In order for magnetic resonance imaging to be applied to endodontic clinical practice, it is necessary to scan at the microscopic level, with microscopy MRI defined as an MRI with voxel resolutions better than 100 mm^3^. Magnetic resonance microscopy chambers are generally small, typically less than 1 cm3. With a resolution of about 100–300 mm, magnetic resonance microscopy could lead to a better understanding of processes that occur inside the teeth.

The obtained microscopic images allow for adequate visualization of the pulp chamber, pulp, and root canals. Ploder et al. used a magnetic resonance exam as an imaging examination complementary to the electrical pulp test for the evaluation of pulp health and of pathological processes occurring within the dental pulp tissue [42]. 

After pulp health determination with the electrical test, healthy pulp could show a signal on T2-weighted images ranged between intermediate and high hyperintense values, which becomes shorter according to patient age, due to secondary dentin accumulation [41,42].

The characteristic of magnetic resonance represents tissues that are rich in water very well, for this reason, the inflammatory response, which develops edema, will be evaluated in an ideal way, and certainly better than dental necrosis, in which we expect a loss in the content of water in the pulp [42].

MRI can therefore be useful in evaluating reperfusion, for example, that concerning regenerative endodontic procedures (REPs) and dental trauma [42,43,44,45].

The application limit of this examination is that, to obtain a sufficient resolution for clinical evaluation in vivo, it takes up to 90 min. It is expected that, with technological development, the imaging time will be reduced in the future, making it fast enough to facilitate clinical use.

The visualization of hard tissues, such as enamel and dentin that do not have MRI signals, considering the low content of protons, remains the real technical challenge to be faced in making MRI a daily diagnostic exam in dentistry [46].

The presented results show the feasibility of using magnetic resonance microscopy to carefully visualize root canal anatomy, applicable for the planning of endodontic procedures while avoiding NiTi rotary instruments, intracanal separation, or other iatrogenic errors, without having an increase in radiation-related biological risks [39,47].

### 4.5. Implantology

Regarding implantology, the aim of the study proposed by Probst et al. was to show whether computer-aided 3D implant planning with template-guided positioning of dental implants based on MRI data is a clinically valid procedure [48]. 

It is very important to point out that all cases in this study were performed by guided implant surgery, virtually planned based on MRI and intraorally transferred by static guides. It is necessary to underline that the authors have reported a deviation between the virtually planned implant position and the resulting final implant position, a deviation of occlusal surfaces between the digitized and occlusal plaster models derived from the MRI data, and the visualization of important anatomical structures that was completely acceptable for clinical application. It is, therefore, possible to define that MR images are sufficiently accurate to show all anatomical structures relevant to dental implant planning, free from ionizing radiation, with an excellent risk/benefit ratio.

In the typical MRI representation, hard dental tissues and bone tissue appear extremely dark due to the poor liquid composition. However, by inverting the dark signal values of the MR image datasets, it is possible to provide a bright or white color to the teeth and various bone structures, and so, an image more similar to CBCT is obtained [12,13,48]. 

The sequence parameters have been optimized considering the spatial resolution and total image acquisition time requirements; therefore, the longer the image acquisition time, the greater the chance of motion artifacts occurring.

The aforementioned authors suggest that the isotropic 3D size with a 0.6 mm3 voxel resulted in a reasonable acquisition time of just over 3:08 min. 

In implantology it is very important to consider the anatomical limitations. For example, the mandibular canal position, an extremely important limitation in the posterior atrophic mandible, is excellently displayed with the use of the T1-weighted 3D sequence.

In the absence of the cortical bone lining the mandibular canal, or in the presence of metal restorations near the inferior alveolar nerve, artifacts can make its location very difficult. This unfortunate event occurs in both T1-weighted sequences and in CBCT imaging [9,48].

However, MR imaging offers a unique advantage and added value through the application of soft tissue contrast in specific sequences. While the T1-weighted sequence is practically a “bone sequence”, and therefore comparable to CBCT imaging, the T2-weighted STIR sequence can work as a “soft tissue and nerve sequence” during implant planning, which allows for direct nerve and blood vessel imaging [19,20,48,49,50].

With increasingly adequate programming software, it will be possible to obtain more information from both sequences in order to improve implant programming, always with an excellent risk/benefit ratio, considering the absence of exposure to ionizing radiation.

One of the most complex problems to be solved is represented by motion artifacts, which can compromise the overall image quality of MR imaging due to the significantly increased examination times compared to CT or CBCT, which represents the major limitation nowadays [5,7,9,48].

This problem could be solved by trying to reduce the examination time, increasing the stability of the patient’s head, and using more effective software to digitally correct these artifacts. However, there is always the problem of artifacts due to the presence of metallic materials, which can affect the representation of important structures when in proximity [9,10,11,12,13,14].

Except for titanium plates and synthesis screws, artifacts due to the presence of metal dental restorations were limited to the occlusal plane area, and therefore minimally limit the implant treatment plan. 

The presence of artifacts of the occlusal plane can represent a limit just when a tooth-supported template-guide is produced only from the MRI exam. Other anatomical structures such as bones, the maxillary sinus, and soft tissues were substantially unaffected, not compromising implant planning at all [48]. 

Artifacts also represent an important problem for CBCT and CT examinations, considering, moreover, the biological damage that these examinations generate.

However, while some materials such as stainless steel and cobalt–chromium alloy are responsible for pronounced artifacts, both in CBCT and in MRI, that may no longer allow for a reasonable diagnosis, the majority of dental materials such as zirconia, amalgams, gold alloys, gold–ceramic crowns, titanium alloys, some composites, and nickel–titanium cause artifacts in a minor way [9,16,17,18,19,20].

An interesting evaluation regarding the article of Probst et al. is that the tooth-supported templates were obtained exclusively with images from MRI, and not from intraoral scanners or other types of imaging or impressions, thus representing a valid alternative, with excellent clinical results.

The 3D comparisons of deviations between MRI reconstructed and scan-derived tooth surfaces, carried out for further evaluation of the methodology, showed acceptable values for clinical application [20,48]. 

The study emphasizes that these results were achieved with a maximum number of 5–6 metal restorations per jaw [48]. 

It must be considered, however, that a tooth that has undergone artifacts can be excluded from the template, placing it on all available nearby ones.

In addition, MRI can provide added diagnostic value due to the excellent soft tissue contrast, which allows, for example, a direct image of peripheral nerve tissue, such as the inferior alveolar nerve, useful for implant planning, as demonstrated in this study [24,49,50]. 

In radiographic imaging, the problem of artefacts is always present, but peri-implant bone defect evaluation, or studies about bone morphology near the implant, are still being carried out [51].

Moreover, radiographic imaging is used for patient follow-up, but always with exposure to a certain dose of ionizing radiation. Precisely from this perspective, magnetic resonance imaging can become an easily repeatable diagnostic test with an excellent risk/benefit ratio. 

In the context of implant surgery, magnetic resonance imaging allows for the detailed measurement of mucosal thickness and can aid in the planning of palatal tissue harvesting to obtain soft tissue augmentation [52].

Despite the various disadvantages that characterize this method, the possibility of being able to perform an examination with a very low risk/benefit ratio is of truly unparalleled value, which must lead to a greater interest in the development of this diagnostic exam.

### 4.6. Maxillary Sinus Diagnosis and Surgery

Regarding the maxillary sinus, the evaluations made by Aktuna Belgin et al. and Dong et al. underlined its importance, showing how it is a structure that can be well studied by MRI, as also pointed out by Panou et al. and Özdemir et al. [53,54,55,56,57].

Successful treatment of sino-nasal disorders, complete knowledge, and correct visualization of the anatomical conditions of the osteomeatal complex and paranasal sinuses is fundamental in head, neck, and oral-maxillofacial surgery. The maxillary sinuses are very interesting in dental clinical practice, and very frequently studied for atrophic jaw rehabilitation [53]. For this reason, it is possible to affirm that they represent both a limitation and a frequent rehabilitation possibility. Knowledge of the anatomical variables of the maxillary sinus is precious to prevent possible accidents and complications in maxillofacial surgery, as well as in the preoperative evaluation in dental implant treatment or in more complex bone regenerations.

Previous studies have also examined volumetric changes in the maxillary sinus; relationships with tooth position; and orthodontic treatment-induced changes such as rapid expansion, septal deviation, and sinus pathologies, as well as examining the differences in the size and anatomy of the maxillary sinus based on age, sex, and race [53,56,57].

Published studies on maxillary sinus volume have produced differing results. Rani et al. found no significant differences in volume (MSV) between the left and right maxillary sinus, and reported that MSV was significantly higher in males than in females [57].

This finding corroborates the findings of the study of Aktuna Belgin et al.; Özdemir et al. and Butaric et al. stated that maxillary sinus development continues into the second and third decade of life in females and males, respectively, with an age-associated decrease in volume occurring after development is completed. All these results have been confirmed by Rani et al. [55,56,57,58].

Compared to CT and CBCT, MRI has fewer metallic artifacts, but with longer exam execution times, and can be used with 3D medical imaging software, allowing for the examination of images obtained in the axial, coronal, and sagittal planes, showing images very similar to that of CBCT, also by the use of specific filters, to increase the contrast between different structures [9,11,12,13].

In maxillary sinus surgery for implant placement, it is important to know and visualize the state of the Schneiderian membrane and any reactive thickening phenomena [59,60]. CBCT allows for visualizing the three-dimensional bone morphology, but the mucosa is poorly defined, despite exposure to ionizing radiation [2,4,5].

In this evaluation, MRI is positioned as a very interesting exam with great margins for improvement, having also demonstrated its usefulness in complete implant planning and in defining the state of health of the maxillary sinus and the Schneiderian membrane for any bone regeneration [60,61].

Moreover, it is necessary to consider that many imaging systems are undergoing considerable changes due to the continuous development of methods that exploit artificial intelligence (AI).

The development of artificial intelligence (AI) technology has proven to be successful in many research fields of medical imaging and various applications of robotic surgery. It can be extremely useful in recognizing landmarks in MRI and optimizing the image produced. In the near future, as with many kinds of software, we expect applications of these technologies to magnetic resonance too, in order to improve and make the use of this interesting diagnostic exam, free from ionizing radiation, more suitable for clinical dental practice [62,63,64].

## 5. Conclusions

With the development of technology, as happened to CT and CBCT in the past, software programs that perform three-dimensional modelling have been introduced in MRI.

As pointed out, the three-dimensional modelling can be excellently applied in the measurement of maxillary sinus volume and Schneiderian membrane thickness, to decide which rehabilitation is the most suitable. 

Moreover, MRI-based computer-assisted implant surgery is demonstrated to be a feasible and accurate procedure, eliminating radiation exposure.

In addition, MRI, compared to the CBCT, better allows for the diagnostic visualization of soft tissues such as the alveolar inferior nerve, which is the most important limitation in the context of posterior mandible rehabilitation.

MRI could become a more common diagnostic exam, both in research and clinical endodontics, providing the possibility to evaluate decay extensions, vitality, and vascularization of the pulp after trauma or after regenerative endodontics; the presence of soft tissue remnants after endodontic procedures or the early detection of missing canals, cracks, and fractures; and precise follow-up of periapical lesions, with the great advantage of avoiding the risk of ionizing radiation damage.

The development of this method can really allow for an improvement in the diagnosis and prognosis of periapical bone lesions.

The main disadvantages of this examination remain the difficult visualization of tissues poor in water, which, however, has proven to be correctable by dedicated software, and can lead to excellent results. Patients suffering from claustrophobia, the presence of devices that prevent the examination from taking place, artifacts from materials and movements, the cost, the lack of availability, and the long examination time represent the disadvantages that will need to be improved in the future.

The results analyzed in this review highlight the potential of MRI for diagnosis in dental clinical practice, without the risk of biological damage from continuous ionizing radiation exposure.

## Figures and Tables

**Table 1:** Dental materials that generate artifacts.

	Materials	Artifacts and Disadvantages
Orthodontics	NiTi arch wires	Major distortions
	Stainless-steel brackets	Major distortions
Endodontics	Resin-based sealer	No distortions
	Gutta-percha	No distortions
Implant and Prostheses	Implants	Major distortions
	Removable prostheses	Major distortions, and possibility of movement
	Gold crowns	No distortions
	Metal crowns	Minor distortions
	Zirconia	Confilicting results
	Ceramic	No distortions
Restorative Dentistry	Glass ionomer cements	Major distortions
	Composite resins	Major distortions
	Polycarboxylate	Minor distortions
	Zinc phosphate-based cement	Minor distortions
	Modified dimethacrylates	Minor distortions
	Amalgam	Minor distortions

**Table 2:** Studies included in the review.

Title	Possible Applications	Year
Magnetic resonance imaging based computer-guided dental implant surgery—A clinical pilot study	Implantology	2020
Evaluation of magnetic resonance imaging for diagnostic purposes in operative dentistry—a systematic review	Endodontics, conservative dentistry, and anatomy	2019
Virtual implant planning and fully guided implant surgery using magnetic resonance imaging—Proof of principle	Implantology	2020
Magnetic resonance imaging artifacts produced by dental implants with different geometries	Implantology	2020
Magnetic resonance imaging in endodontics: a literature review	Endodontics	2017
Magnetic resonance imaging artefacts and fixed orthodontic attachments	Orthodontics (artefacts)	2015
Human tooth and root canal morphology reconstruction using magnetic resonance imaging	Endodontics, anatomy	2015
MRI for Dental Applications	Endodontics, oral surgery, anatomy	2018
Nuclear Magnetic Resonance Imaging in Endodontics: A Review	Endodontics, conservative denstistry, anatomy, oral surgery	2018
Magnetic resonance imaging in zirconia-based dental implantology	Implantology	2014
High-resolution dental MRI for planning palatal graft surgery—a clinical pilot study	Surgery	2018
Correlation between magnetic resonance imaging and cone-beam computed tomography for maxillary sinus graft assessment	Surgery, maxillary sinus, implantology	2020
Differentiation of periapical granulomas and cysts by using dental MRI: a pilot study	Surgery, endodontics	2018
Assessment of signal-to-noise ratio and contrast-to-noise ratio in 3 T magnetic resonance imaging in the presence of zirconium, titanium, and titanium-zirconium alloy implants	Surgery, implantology	2019
Dental Materials and Magnetic Resonance Imaging	Artefacts	1991
Differential diagnosis between a granuloma and radicular cyst: Effectiveness of Magnetic Resonance Imaging (MRI)	Surgery, endodontics	2018
Unwanted effects due to interactions between dental materials and magnetic resonance imaging: a review of the literature	Artefacts	2018
Accuracy and Reliability of Root Crack and Fracture Detection in Teeth Using Magnetic Resonance Imaging	Endodontics, conservative dentistry	2019
Magnetic Resonance Imaging in Endodontic Treatment Prediction	Endodontics	2010
The value of the apparent diffusion coefficient calculated from diffusion-weighted magnetic resonance images in the differentiation of maxillary sinus infiammatory diseases	Maxillary sinus	2018
Season, Age and Sex-Related Differences in Incidental Magnetic Resonance Imaging Findings of Paranasal Sinuses in Adults	Maxillary sinus	2019
Anatomical variation in maxillary sinus ostium positioning: implications for nasal-sinus disease	Maxillary sinus	2018
Metal-induced artifacts in MRI	Artefacts	2011
Protocol for the Evaluation of MRI Artifacts Caused by Metal Implants to Assess the Suitability of Implants and the Vul-nerability of Pulse Sequences	Artefacts	2018
Influence of magnetic susceptibility and volume on MRI artifacts produced by low magnetic susceptibility Zr-14Nb alloy and dental alloys	Artefacts	2019
Dental MRI using a dedicated RF-coil at 3 Tesla	Artefacts	2015
Artifacts in magnetic resonance imaging and computed tomography caused by dental materials	Artefacts	2012
Evaluation of magnetic resonance imaging artifacts caused by fixed orthodontic CAD/CAM retainers-an in vitro study	Artefacts,	2012
Artifact Properties of Dental Ceramic and Titanium Implants in MRI	Artefacts	2018
PETRA, MSVAT-SPACE and SEMAC sequences for metal artefact reduction in dental MR imaging	Artefacts	2017
Magnetic resonance imaging in zirconia-based dental implantology	Artefacts, implantology	2015
Assessment of apical periodontitis by MRI: a feasibility study	Surgery, endodontics	2015
Magnetic Resonance Imaging in Endodontic Treatment Prediction	Endodontics	2011
Ultrashort echo time (UTE) MRI for the assessment of caries lesions	Endodontics, conservative dentistry	2013
Reperfusion of autotransplanted teeth--comparison of clinical measurements by means of dental magnetic resonance im-aging	Endodontics, surgery	2013
Early detection of pulp necrosis and dental vitality after traumatic dental injuries in children and adolescents by 3-Tesla magnetic resonance imaging	Endodontics	2015
Optimized 14 + 1 receive coil array and position system for 3D high-resolution MRI of dental and maxillomandibular structures	Endodontics	2016

## Data Availability

Not applicable.
